# Unveiling the potential benefits of statins in medulloblastoma treatment: A promising therapeutic approach

**DOI:** 10.1016/j.ibneur.2025.08.016

**Published:** 2025-08-20

**Authors:** Amir Modarresi Chahardehi, Aida Naseri, Esfandiar Mali, Leili Ziaei, Fatemeh Moradi, Fatemeh Teimourpour, Leila Tajik, Hossein Motedayyen, Mohammad Saeed Soleimani Meigoli, Reza Nasiri, Reza Arefnezhad, Fatemeh Rezaei-Tazangi

**Affiliations:** a Student Research Committee, Fasa University of Medical Sciences, Fasa, Iran; b Faculty of Medicine, Shiraz University of Medical Sciences, Shiraz, Iran; c Department of Pediatrics, Shiraz University of Medical Sciences, Shiraz, Iran; d Faculty of Medicine, Islamic Azad University Tehran Medical Sciences, Tehran, Iran; e Faculty of Medicine, Mashhad University of Medical Sciences, Mashhad, Iran; f Department of Biology, Science and Research Branch, Islamic Azad University, Tehran, Iran; g Autoimmune Diseases Research Center, Kashan University of Medical Sciences, Kashan, Iran; h Faculty of Medicine, Fasa University of Medical Sciences, Fasa, Iran; i School of Medicine, Shiraz University of Medial Sciences, Shiraz, Iran; j Student Research Committee, Shiraz University of Medical Sciences, Shiraz, Iran; k Department of Anatomy, School of Medicine, Fasa University of Medical Sciences, Fasa, Iran

**Keywords:** Medulloblastoma, Statin, Radiation, Chemotherapy

## Abstract

Medulloblastoma (MB) is the predominant malignant brain tumor in children, known for its tendency to spread and reoccur. Although current treatment methods, which include surgery, chemotherapy, and radiation, are used in combination, the chances of survival are not as great as desired, especially for individuals at high risk. Moreover, traditional treatments are linked to significant and lasting neurocognitive and endocrine adverse reactions, highlighting the necessity for more efficient and less harmful treatment approaches. Statins, which are commonly used drugs for reducing cholesterol levels, have received much interest because of their pleiotropic effects, which may include possible anticancer qualities. Preclinical investigations have shown that statins can hinder the growth and multiplication of MB cells by targeting the mevalonate system, triggering apoptosis, and regulating cellular signaling pathways related to carcinogenesis. Statins have demonstrated potential in specifically targeting the Sonic Hedgehog (SHH) subgroup of MB, characterized by abnormal activation of the Hedgehog signaling system. Moreover, recent evidence indicates that statins with traditional chemotherapeutic drugs or targeted therapies can enhance their anticancer effects and overcome resistance mechanisms. This study thoroughly debates the possible advantages of statins in treating MB, including their mechanisms of action and the implications for future therapeutic approaches. This review examines the impact of statins on tumor growth, apoptosis induction, chemosensitivity enhancement, and tumor recurrence reduction. It emphasizes the potential of repurposing these well-established medications for treating this severe pediatric cancer.

## Highlights

• Medulloblastoma (MB) is the predominant malignant brain tumor in children, known for its tendency to spread and reoccur.
• Statins have received much interest because of their pleiotropic effects.
• Preclinical investigations have shown that statins can hinder the growth and multiplication of MB cells by targeting the mevalonate system, triggering apoptosis, and regulating cellular signaling pathways related to carcinogenesis.
• Statins have demonstrated potential in specifically targeting the Sonic Hedgehog (SHH) subgroup of MB.
• Statins with traditional chemotherapeutic drugs or targeted therapies can enhance their anticancer effects and overcome resistance mechanisms.

## Graphical Abstract

**Figure:**
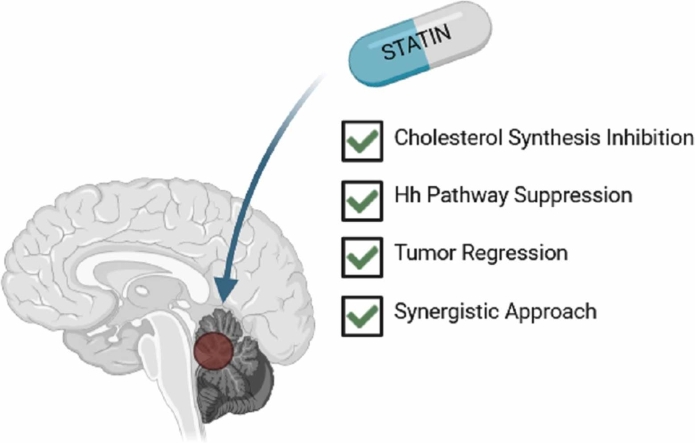

## 1 Introduction

Medulloblastoma (MB) is a heterogeneous, invasive, and highly aggressive central nervous system (CNS) malignancy widely recognized as the most prevalent pediatric solid tumor (Gwynne et al., 2022, Mduma et al., 2022, Jackson and Packer, 2023, Fang et al., 2022). It is frequently observed in males, with a median age of 7 years (Le Teuff et al., 2020). Originating mostly from the cerebellar vermis and localized near the fourth ventricle (Aljoghaiman et al., 2018, Kabir et al., 2020, de Oliveira et al., 2018), MB accounts for approximately 25–30 % of pediatric brain tumors and nearly 40 % of childhood tumors in the posterior fossa (Fang et al., 2022). Initially considered a single disease, the advent of genome-wide sequencing has enabled meticulous molecular classification, revealing four distinct subgroups: Sonic Hedgehog (SHH), WNT-activated, group 3, and group 4, each characterized by unique molecular alterations, demographics, origins, and clinical outcomes (Wen and Hadden, 2021, Menyhart and Gyorffy, 2020). Although these general subgroups remain the same during relapse, their transcriptomes frequently change, potentially determining the location and timing of tumor recurrence (Okonechnikov et al., 2023, Ramaswamy et al., 2013). For instance, a retrospective study of 200 adult MB patients in the United States revealed that approximately 40 % of recurrences, presenting as leptomeningeal or distant metastases, occurred distant from the primary tumor site (Gregory et al., 2023). The strategic positioning of MB induces symptoms such as vomiting, nausea due to chemoreceptor trigger zone (CTZ) stimulation, increased intracranial pressure (ICP) leading to lethargy, irritability, loss of appetite, morning headaches, behavioral changes, and cerebellar dysfunction manifesting as gait ataxia, extremity disturbances, and poor coordination (Franceschi et al., 2021, Rawal et al., 2020). With a potential for metastasis through cerebrospinal fluid (CSF) pathways, 30 % of cases present with metastatic disease at diagnosis, warranting proper therapeutic strategies. Considering its expected outcome without treatment, MB has been classified as a WHO grade IV, the highest in malignant tumors. Therefore, proper therapeutic strategies are required (Cotter and Hawkins, 2022, Martin-Rubio et al., 2022). The current multidisciplinary treatment approach for MB typically involves surgical resection followed by radiotherapy and chemotherapy; however, treatment sequencing may vary based on patient age and risk stratification, such as the use of neoadjuvant chemotherapy in infants. Despite significantly improving the 5-year survival rate, recurrence and metastasis remain the primary causes of death. Moreover, the risk of toxicity is considerable in children under three years old and infants, often resulting in severe endocrine and neurological deficits (Kabir et al., 2020, Menyhart and Gyorffy, 2020, Zhou et al., 2023, Zhang and Wang, 2021, Gajjar et al., 2021). Each component of the therapy contributes to complications, leading to severe and chronic health conditions (Menyhart and Gyorffy, 2020). Notably, the current strategy may not be equally effective for all MB subgroups, with studies indicating a 90 % survival rate for WNT cases but less than 50 % for group 3 patients (Cotter and Hawkins, 2022, Ray et al., 2021). Consequently, the main strategy for novel anti-MB drugs aims to selectively target the abnormal genes and proteins specific to each subgroup (Wen and Hadden, 2021).

The Mevalonate (MV) pathway, also dubbed the cholesterol synthesis route, serves as a vital regulator in maintaining the delicate equilibrium of cellular metabolism. Statins, frequently prescribed medications, operate by impeding the key enzyme as a 3-hydroxy-3-methylglutaryl coenzyme A (HMG-CoA) reductase (HMGCR) along this pathway, thus reducing cholesterol levels (Tripathi et al., 2024). A succession of epidemiological investigations into statin drugs, which are utilized for lowering cholesterol, have showcased their ability to deter the recurrence of various types of cancer (Kimura et al., 2023). The anticancer properties of statins have been extensively investigated in various *in vitro* and *in vivo* studies (Alizadehasl et al., 2024), providing a robust research base that underscore their potential in cancer treatment. Hence, statins, as HMG-CoA reductase inhibitors, constitute a class of medications primarily used to manage hypercholesterolemia and prevent cardiovascular diseases (Morofuji et al., 2022). Initially designed to impede cholesterol biosynthesis, statins have garnered considerable interest due to their pleiotropic effects, including potential anticancer activity (Ahmadi et al., 2020). Multiple proposed mechanisms have been implicated in the antitumor effects of statins, encompassing the inhibition of cell proliferation, induction of apoptosis, suppression of angiogenesis, and modulation of cellular signaling pathways (Zaky et al., 2023). Furthermore, statins can cause cancer cells to undergo oxidative stress, cell cycle arrest, and autophagy. Interestingly, clinical trials have linked statin usage to a reduced malignancy risk, lower grade upon diagnosis, lower chance of local recurrence, and improved survival rates (Zaky et al., 2023). Statins are inhibitors of HMG-CoA reductase (HMGCR), the rate-limiting enzyme in the mevalonate pathway responsible for cholesterol biosynthesis (Patel et al., 2022), have demonstrated promising therapeutic effects against various cancers. From breast cancer (Filaferro et al., 2024) and prostate cancer (Gobel et al., 2024) to colorectal cancer (Loffler et al., 2024) and glioma (Alrosan et al., 2023), statins have shown anticancer effects in a plethora of preclinical investigations. The Hedgehog (Hh) signaling pathway plays a crucial role in cell patterning and differentiation during embryonic development, and its dysfunction is implicated in various disorders, including MB (Tang and So, 2007, Jiang, 2022). The SHH subgroup of MB (SHH-MB) accounts for nearly 30 % of cases. It is characterized by abnormal activation of the Hh pathway due to exacerbations in Gli1, Gli2, N-Myc, or mutations in Smo, Sufu, and Ptch1 (Jiang, 2022, Fan et al., 2021). On the other hand, cholesterol is a vital component in synthesizing the Shh ligand, suggesting the importance of sterol synthesis in Hh signaling (Tang and So, 2007, Corcoran and Scott, 2006). Statins primarily impede the Hh pathway by inhibiting cholesterol synthesis, which is essential for the genetic activation leading to MB (Fan et al., 2021). Studies have demonstrated that statins repress the Hh pathway in MB cells and fibroblasts, suppressing tumor growth and proliferation (Gordon et al., 2018). Additionally, simvastatin has been shown to induce apoptosis in both SHH-MB and Group 3/4 MB by inhibiting the MV pathway (Sheikholeslami et al., 2019). Furthermore, the investigation on animals and cells has indicated statins to have a considerable role in therapeutic strategies for CNS cancers; hence, the quest of whether statins can also treat MB has been continued (Tang and So, 2007).

Building on the accumulating evidence of statin efficacy in various malignancies, this review explores the potential of statins as a therapeutic strategy for MB. The focus lies on the potential benefits of statins for improving treatment outcomes, including overall survival and quality of life.

## 2 Pathogenesis of medulloblastoma

As previously mentioned, MB is the most common malignant brain tumor in children, constituting roughly 20 % of all pediatric CNS tumors (Smoll and Drummond, 2012). It originates from undifferentiated cells within the cerebellum, previously thought to be derived from the medulloblast near the fourth ventricle's ependymal lining (Ferguson and Lesniak, 2005). While any embryonal brain tumor in the cerebellum, cerebellar peduncle, or fourth ventricle suggests MB, rare similar-appearing malignant tumors can occur in this region (Sturm et al., 2016). The pathogenesis of MB remains a complex and fascinating scientific question. Current understanding suggests that granule cell precursors originating from the developing cerebellum's external germinal layer (EGL) contribute to tumor growth within the fourth ventricle. The tumor can then enlarge in this location, spreading to the cerebellar vermis and brainstem and potentially seeding along the craniospinal axis. MB exhibits aggressive behavior, characterized by local tissue invasion and a propensity for metastasis via the subarachnoid space throughout the brain and spinal cord (Raffel, 2004).

Historically, MB classification encompassed both molecular and histological features (Kool et al., 2012). In 2016, the World Health Organization (WHO) classification revised its MB classification system based on advancements in understanding the tumors' genomics, biology, and clinical behavior (Louis et al., 2021). The most recent WHO classification of MB provides two main categories: genetically-defined and histologically-defined (Louis et al., 2016).

1. The genetically-defined classification system includes WNT-activated, SHH-activated TP53 wildtype, SHH-activated TP53-mutant, and non-WNT/non-SHH.
2. The histologically-defined classification system, a key component in the diagnosis of MB, includes classic, desmoplastic/nodular (DN), large cell/anaplastic (LCA), and medulloblastoma with extensive nodularity (MBEN) (Cotter and Hawkins, 2022). The MBEN category can be further divided into subclasses, known as group 3 and group 4 if differentiation is required.

Finally, the category "MB, NOS (not otherwise specified)" is used when a MB tumor cannot be definitively classified within either the genetic or histological systems (Louis et al., 2016).

### 2.1 Histologically-defined classification

MB exhibits histological heterogeneity, with distinct variants classified based on their morphology. The most common variant is the classic type, accounting for 72 % of cases (Louis et al., 2016). The presence of characterized classic MBs:

• Small round blue cells
• Absence of other defining features such as reticulin-invested nodules or cytologic anaplasia
• Well-defined Homer Wright rosettes, observed in only a subset of classic MBs (Cotter and Hawkins, 2022).

Desmoplastic/nodular (DN) variant and MBEN are two other notable histological subtypes. These variants are characterized by:

• Nodularity with neurocytic differentiation, surrounded by embryonal element
• Potential presence of desmoplasia, indicated by collagen deposition around cells (Orr, 2020)
• Reticulin staining, a crucial diagnostic tool for identifying these subtypes
• Increased Ki-67 labeling in the internodular regions, a marker of cell proliferation (Cotter and Hawkins, 2022)

It is important to note that MBs involving the subarachnoid space can also exhibit desmoplastic features (Cotter and Hawkins, 2022).

MBEN represents a distinct subtype within the DN variant. These tumors tend to have a more central location compared to classic DN tumors and are typically diagnosed in infants. A defining feature of MBEN is the presence of irregular-shaped nodules, often with a merging pattern, instead of the primitive elements commonly observed in classic MB. Another hallmark characteristic is the "streaming" pattern, where linear arrangements of neurocytic cells interconnect neighboring nodules (Orr, 2020). Interestingly, approximately 50 % of DN cases and around 20 % of MBEN cases are diagnosed in children under three. Early childhood diagnosis of these subtypes is associated with a more favorable prognosis (Cotter and Hawkins, 2022).

The large cell/anaplastic (LC/A) variant is characterized by enlarged cells with prominent nucleoli. Anaplastic features may or may not be present, such as significant pleomorphism, nuclear enlargement, and disrupted cell-cell connections. Classification as LC/A requires anaplastic features to be evident in most tumor samples. This variant typically exhibits a high mitotic and apoptotic rate. LC/A MB is frequently diagnosed in patients with established metastatic disease (60–70 %) and demonstrates a high propensity for relapse and spread via the cerebrospinal fluid. Notably, large cells with prominent nucleoli are often associated with amplifying the MYC or MYCN oncogenes (Cotter and Hawkins, 2022).

### 2.2 Genetically-defined classification

**WNT-Activated Variant:** WNT-activated MB is a distinct subtype characterized by its origin close to the midline structures of the brain. These tumors can also involve the cerebellar peduncle and brainstem, extending through the Foramen of Luschka (Patay et al., 2015). As mentioned above, this variant primarily affects older children, typically over four years of age, and constitutes approximately 10 % of all MB cases (Fang et al., 2022, Juraschka and Taylor, 2019). WNT-activated MB exhibits a unique association with:

• **Increased bleeding:** This subtype is associated with significant bleeding events due to blood-brain barrier permeability and underdevelopment (Phoenix et al., 2016).
• **Cellular origin:** Transcriptional profiling suggests that WNT-activated MB may originate from precursor cells located in the dorsal brainstem (Mir et al., 2017).

**SHH-activated variant:** SHH-activated MB arises predominantly in the cerebellar hemisphere, although involvement of the cerebellar vermis also occurs (Franceschi et al., 2021). This subtype is believed to originate from a population of external granule cell neurons within the cerebellum. A key differentiating factor within this variant is the presence or absence of TP53 mutations (Rawal et al., 2020).

SHH-activated MB presents a unique challenge in its therapeutic management. It exhibits a bimodal distribution in age at diagnosis, with a first peak observed in infants and a second peak in adults and children over 16 years old (Louis et al., 2016). The primary therapeutic targets within the SHH pathway include antagonists of the Smoothened (SMO) receptor and inhibitors of the GLI1 transcription factor. However, the SHH subtype poses a significant challenge due to its impenetrable blood-brain barrier, which hinders the passage of both small and large therapeutic molecules such as chemotherapy drugs (Fang et al., 2022). This unique characteristic of the SHH subtype underscores the need for innovative therapeutic approaches.

**Fig. 1:**
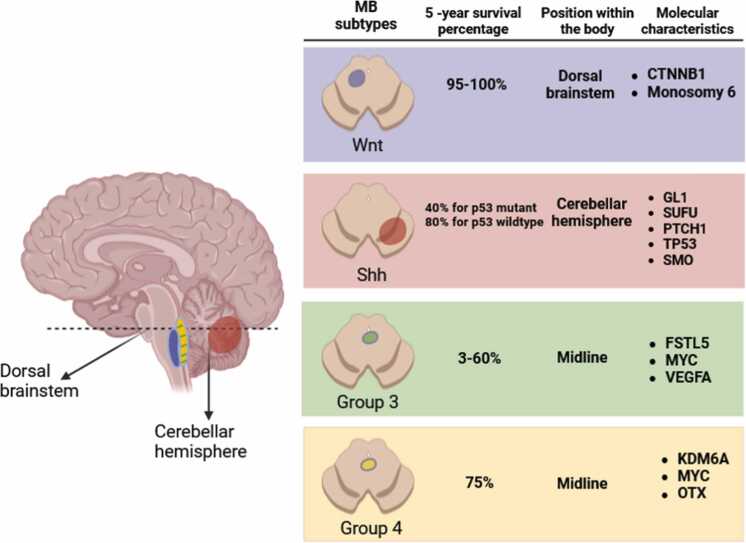
Medulloblastoma subtype location, prognosis, and molecular characteristics (adapted from Fang et al., 2022) (Fang et al., 2022). This Figure is designed with BioRender.com.

**Non-WNT/Non-SHH variants (Groups 3 and 4):** Comprising the largest proportion of MB cases, the non-WNT/non-SHH subgroup encompasses tumors that do not exhibit activation of the WNT or SHH signaling pathways. These subgroups account for most MB cases (Cotter and Hawkins, 2022). Non-WNT/non-SHH MBs, typically located in the brain's midline and often filling the fourth ventricle, present intriguing challenges during treatment due to their specific clinical presentations and the implications of their localization (Martin-Rubio et al., 2022). Medulloblastoma exhibits distinct molecular subtypes distinguished by their distinctive molecular and anatomical characteristics, as depicted in Fig. 1.

## 3 Benefits of statins in medulloblastoma treatment

Preclinical studies suggest that statins impede MB cell growth and proliferation through multiple mechanisms. By inhibiting the MV pathway, statins deplete intracellular pools of isoprenoid intermediates like farnesyl pyrophosphate (FPP) and geranylgeranyl pyrophosphate (GGPP), essential for proper localization and function of signaling proteins and small GTPases involved in cell cycle progression and survival (Waller et al., 2019, Gbelcova et al., 2017). Interestingly, statins exhibit dose-dependent effects on angiogenesis, inhibiting it at higher doses and potentially promoting it at lower doses (Santoni et al., 2022).

Statins, with their potential to overcome resistance mechanisms, offer a promising avenue for research in targeting the SHH subgroup of MB. This subgroup is characterized by the aberrant activation of the Hh signaling pathway (Gordon et al., 2018). Statins, by effectively suppressing the Hh pathway through the inhibition of cholesterol biosynthesis, have shown to reduce tumor growth and proliferation in SHH-driven MB (Xiao et al., 2017). Statins effectively suppress the Hh pathway by inhibiting cholesterol biosynthesis, thereby reducing tumor growth and proliferation in SHH-driven MB (Fan et al., 2021). While preclinical studies have primarily focused on statins as monotherapy, emerging evidence suggests their potential to be synergistically combined with other therapeutic modalities to overcome resistance mechanisms and enhance efficacy (Liu et al., 2023). Notably, statins have shown promise in potentiating the cytotoxic effects of chemotherapeutic drugs, radiation therapy, and targeted therapies in various cancer models (Altwairgi, 2015).

### 3.1 Anti-tumor effects

**Fig. 2:**
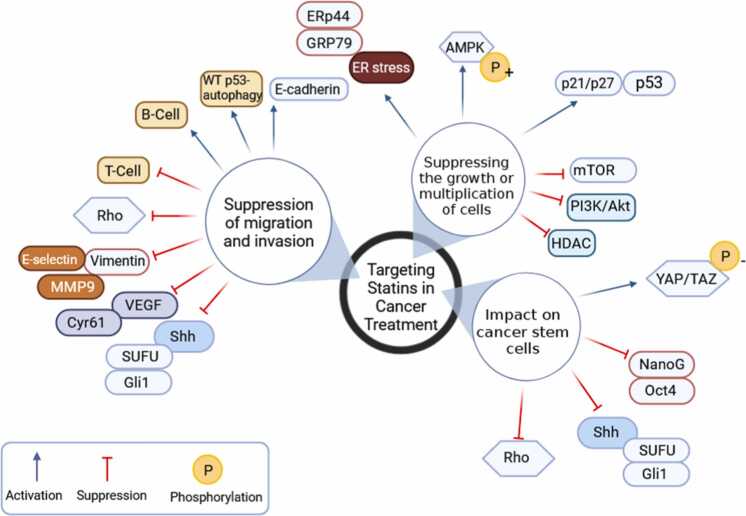
A review of the primary molecular targets of statins in relation to cancer (adapted from Di Bello et al. 2020) (Di Bello et al., 2020). This Figure is designed with BioRender.com.

Beyond their cholesterol-lowering effects, statins demonstrate pleiotropic properties relevant to cancer therapy, including inducing chemotherapy sensitivity, promoting apoptosis, and inhibiting tumor cell proliferation and invasion—these characteristics position statins as potential therapeutic candidates for MB. Notably, statins exert their primary anti-tumor effects by targeting the MV pathway. However, their influence extends beyond cholesterol biosynthesis, impacting various cell death pathways and potentially reprogramming the tumor microenvironment (TME) (Khalaf et al., 2021). For instance, according to Gordon et al. using statins to limit cholesterol production repressed the Hh pathway in fibroblasts and MB cells, decreasing tumor development and MB proliferation (Gordon et al., 2018). The primary molecular targets of statins in cancer are summarized in Fig. 2.

Statins have an antiproliferative impact on cancer cells by raising ER stress indicators GRP78 and ERp44 levels, phosphorylating S6 and 4EBP1, blocking the mTOR pathway, and inhibiting HDACs. Statins activate the LKB1-AMPK-p38MAPK-p53 survivin signaling cascade in cancer cells by phosphorylation and acetylation of p53 (Okubo et al., 2020, Huang et al., 2020). Also, statins stimulate T-cell suppression and B-cell survival and enhance autophagy that depends on the WT p53 protein, preventing migration and invasion (Chou et al., 2019). In addition, statins can decrease the expression of Shh in cancer stem cells (CSC), which in turn leads to a reduction in the movement and infiltration of cancer cells (Yin et al., 2018).

#### 3.1.1 Mevalonate pathway and medulloblastoma therapy

**Fig. 3:**
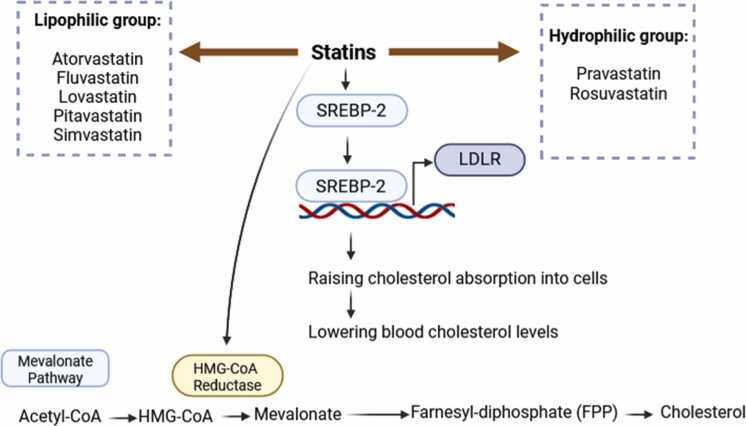
Statin function and various types (adapted from Vuu et al., 2023) (Vuu et al., 2023). HMG-CoA: 3-hydroxy-3-methyl-glutaryl-coenzyme A; LDLR: low-density lipoprotein receptor; SREBP-2: sterol regulatory element-binding protein 2. This Figure is designed with BioRender.com.

The MV pathway, a critical metabolic process for maintaining membrane integrity, cell signaling, and protein synthesis (Fan et al., 2021), can be effectively targeted for cancer treatment using statins. It also leads to the production of isoprenoids, including cholesterol and others with diverse biological functions (Lasuncion et al., 2022). Statins, known for their therapeutic role in cardiovascular health, exert their anti-tumor effects by specifically targeting cholesterol-dependent and independent steps within the MV pathway (Fan et al., 2021, Jiang et al., 2021). By inhibiting HMGCR, the rate-limiting enzyme in cholesterol synthesis, statins impede protein prenylation, a crucial process for the function of various signaling proteins involved in tumorigenesis and drug resistance (Göbel et al., 2020, Konstantinopoulos et al., 2007). Acetyl-coenzyme A (CoA), the final product of glycolysis, kick-starts the MV cascade, converting subsequently into HMG-CoA via the enzyme HMGCR, thus initiating mevalonate synthesis. For example, a study by Sheikholeslami et al. found that simvastatin, being lipophilic in nature, acts as a drug that inhibits HMG-CoA reductase and exhibits pleiotropic effects. By blocking HMG-CoA reductase, it halts the production of crucial downstream intermediates in the mevalonate cascade, including FPP and GGPP (Sheikholeslami et al., 2019). This mevalonate molecule further metabolizes into FFP, a critical precursor in the biosynthesis of sterols like cholesterol. These lipids and GGPP, a downstream product of FFP, are crucial components for post-translational modifications in proteins (Guerra et al., 2021). These modifications, including protein prenylation (attachment of geranylgeranyl or farnesyl isoprenoid to proteins) and N-glycosylation (conjugation of proteins' asparagine residues with glycans), facilitate various cellular processes (Palsuledesai and Distefano, 2015, Breitling and Aebi, 2013). Notably, GGPP and FFP enable the conjugation of proteins, notably oncogenic Ras, Rac, and Rho GTPases, thereby influencing cellular signaling pathways. Previous investigations have discussed the control of mevalonate and related metabolic pathways as potential strategies for preventing cancer and intervening in treatment pathways (McGregor et al., 2020). These studies demonstrate a correlation between heightened activity in the mevalonate pathway and the advancement of cancer. Consequently, targeting and interfering with these pathways, particularly with statins, is regarded as a viable technique for treating cancer (Wong and Fatehi Hassanabad, 2021). Statins suppress the activity of HMG-CoA reductase, an enzyme that controls the pace of a biochemical process called the mevalonate pathway. This system is responsible for the synthesis of cholesterol in the liver. Statins also enhance the production of SREBP-2, which acts as a transcription factor for several genes involved controlling lipid balance, including LDLR. LDLR on cell membranes enhances cholesterol uptake into hepatocytes and other cells, hence aiding in the reduction of plasma cholesterol levels (Fig. 3) (Vuu et al., 2023).

Mevalonate's role in de novo cholesterol synthesis is crucial for tumorigenesis (Jiang et al., 2021). Statins, as we know, target the MV pathway by inhibiting HMGCR, the rate-limiting enzyme, and upregulating the LDL receptor (LDLR), consequently lowering serum LDL levels and cholesterol production. This reduction in cholesterol disrupts lipid rafts, specialized membrane domains enriched in sphingolipids and cholesterol that are essential for signaling receptor activation. Consequently, statin-mediated disruption of lipid rafts impedes key cellular processes in cancer cells, including proliferation, survival, and metastasis (Liu et al., 2023, Li et al., 2022).

#### 3.1.2 Inhibition of Hedgehog pathway

Statins, by suppressing the Hh signaling pathway, show significant potential in exerting anti-tumor effects in MB (Fan et al., 2021). Cholesterol, a critical component for proper Smoothened (Smo) protein function, serves as an essential element in the Hh pathway. Statin therapy, by reducing cellular cholesterol levels, indirectly disrupts Hh signaling, thereby hindering tumor cell proliferation and survival (Gordon et al., 2018). This pathway's dynamics involve the interplay of transmembrane proteins, Patched (Ptc) and Smo. In the absence of the Hh ligand, Ptc acts as a repressor, inhibiting Smo's signaling activity and preventing the activation of downstream Hh target genes (Guerra et al., 2021). Notably, Fan et al. (2021) demonstrated that oral simvastatin administration at 100 mg/kg effectively suppressed Hh signaling and reduced MB tumor size in mice without compromising bone development. This finding underscores the potential of statins, including simvastatin and atorvastatin, to curb MB growth while preserving bone health in preclinical models (Fan et al., 2021).

#### 3.1.3 Induction of apoptosis

Studies demonstrate the pro-apoptotic properties of statins in MB cells, contributing to their potential for directly reducing tumor cell viability and enhancing the effectiveness of chemotherapy (Vuu et al., 2023). This apoptotic effect, which is demonstrably independent of Hh pathway inhibition, has been observed in various MB cell lines in a time- and dose-dependent manner. Importantly, statins show a unique ability to induce apoptosis selectively in tumor cells, while sparing normal cells. This differential effect offers a potentially favorable therapeutic index, a crucial consideration in pediatric MB treatment where minimizing side effects is a priority (Sheikholeslami et al., 2019). Furthermore, both clinical and *in vitro* evidence suggest a synergistic effect of statins when combined with conventional chemotherapeutic agents. For instance, studies in glioblastoma multiforme, a brain tumor with similarities to MB, have shown that the combination of simvastatin with chemotherapy significantly reduces tumor growth and migration. These findings highlight the potential for similar synergistic effects in MB therapy, warranting further investigation into the underlying mechanisms (Vuu et al., 2023).

Apoptosis, orchestrated by the B-cell lymphoma 2 (Bcl-2) family via the intrinsic pathway and the tumor necrosis factor (TNF) receptor superfamily through the extrinsic pathway, plays a pivotal role in this process (Singh and Lim, 2022, Roberts et al., 2022). Studies have shown that simvastatin can induce apoptosis in MB cells through caspase-dependent and caspase-independent pathways. Sheikholeslami *et al*. investigated the latter pathway, elucidating simvastatin's impact on the expression of Bcl-2, Bcl-xl, and Bax (Sheikholeslami et al., 2019). Similarly, Fan *et al*. confirmed the involvement of statins in programmed cell death through the activation of caspases (Fan et al., 2021). Ferroptosis is a form of iron-dependent cell death characterized by lipid peroxidation and regulated by glutathione peroxidase 4 (GPX4) activity. Statins can promote ferroptosis by inhibiting HMGCR and reducing GPX4 activity (Sahebkar et al., 2023, Bebber et al., 2020). This mechanism offers the potential for selective tumor cell eradication independent of the immune system. However, ferroptosis can exhibit paradoxical effects, promoting tumor growth or immune evasion under certain conditions. Yao *et al*. designed a nanomedicine containing simvastatin and demonstrated its ability to induce ferroptosis in triple-negative breast cancer (TNBC) by reducing GPX4 levels (Yao et al., 2021). Given the role of lipid peroxidation in brain tumors and the potential benefits of ferroptosis, statins warrant investigation for their ability to induce ferroptosis in MB (Jaganjac et al., 2021). Importantly, the safety and efficacy of statins in MB require thorough evaluation across various models, considering the unique properties of this childhood brain tumor and the potential drawbacks associated with ferroptosis.

Pyroptosis, a recently discovered form of programmed cell death characterized by DNA fragmentation, cell swelling, and the release of proinflammatory mediators like interleukin (IL)-1β, IL-18, and IL-1α, has emerged as a potential target in cancer therapy (You et al., 2023). Mutations in DDX3X are common in certain subgroups of medulloblastoma, and the expression of DDX3X mutants enhances Wnt pathway signaling. In rodent models of medulloblastoma, knockout of DDX3X increased the likelihood of disease development and shortened the time it takes for tumors to form. This effect was driven by the activation of inflammasomes and cell pyroptosis (Patmore et al., 2020). Gasdermin proteins, particularly gasdermin D (GSDMD), drive pyroptosis, with proinflammatory caspases (e.g., caspases-1/4/5/11) contributing to the cleavage and activation of these pore-forming proteins (McKenzie et al., 2020). Pyroptosis can occur through canonical and noncanonical pathways, and recent literature suggests that statins may suppress pyroptosis by modulating oxidative stress and blocking the canonical pathway (Khalifeh et al., 2021). Recent literature indicates that statins suppress pyroptosis by intervening in oxidative stress and blocking the canonical pathway (Filaferro et al., 2024). *In vivo* and *in vitro* experiments of Wang *et al*. demonstrated that simvastatin activated caspase-1, IL-1β, and IL-18, hence triggering pyroptosis in lung cancer (Gobel et al., 2024). However, further investigation is necessary to determine the potential impact of statin-mediated pyroptosis regulation in MB, as excessive pyroptosis can lead to detrimental tissue damage due to an overactive immune response (Filaferro et al., 2024).

#### 3.1.4 Tumor microenvironment (TME)

The TME is a complex ecosystem that fosters tumor cell proliferation and plasticity through dynamic cell-cell and cell- extracellular matrix (ECM) interactions (Khalaf et al., 2021). In MB, the TME is a composite of various cells (neurons, astrocytes, microglia) and ECM components, influencing tumor initiation, progression, and recurrence (van Bree and Wilhelm, 2022). Statins exert their anticancer effects by reprogramming the acidic, immune, anoxic, and metabolic microenvironments within tumors. Firstly, they mitigate the disruption of the acidic microenvironment by reducing levels of free fatty acids in the blood, which are major contributors to the elevated synthesis of fatty acids and accumulation of H+ ions within the TME. Secondly, statins target the immune microenvironment, which is often characterized by high cholesterol levels and immunosuppressive signals. Cholesterol upregulation can impair the function of CD8 + T-lymphocytes (van Bree and Wilhelm, 2022) leading to their exhaustion via inhibitory receptors like programmed death-1 (PD-1). Statins promote T-cell function by downregulating PD-L1 expression on tumor cells, thereby enhancing T-cell-mediated anti-tumor immunity. This mechanism enhances T-cell-mediated anti-tumor immunity (Choe et al., 2022), thus enhancing their ability to combat cancer (Ando and Araki, 2022). In the context of MB, statins have been found to specifically target the immune microenvironment, leading to a reduction in immunosuppressive signals and an enhancement of T-cell-mediated anti-tumor immunity.

Statins, by targeting the hypoxic microenvironment, a key contributor to tumor progression, induce the activation of AMPK. The activation counteracts hypoxia-inducible factor-1α (HIF-1α) signaling, thereby inhibiting the production of pro-angiogenic factors (van Bree and Wilhelm, 2022). This effect of statins may translate to improved efficacy of PD-1 blockade therapy, as demonstrated by Kansal *et al*. in preclinical models of head and neck squamous cell carcinoma (HNSCC). Furthermore, statins can modulate the tumor's immune response. Studies suggest that statins promote a shift from M2 to M1 macrophages and enhance T-lymphocyte cytotoxicity, potentially potentiating their anti-tumor effects (Kansal et al., 2023). Similarly, Pokhrel *et al*. found that simvastatin's anticancer effects were potentiated through intraperitoneal injection, further highlighting its potential as an adjuvant therapy (Pokhrel et al., 2021). Yes-associated protein (YAP) and transcriptional coactivator with PDZ-binding motif (TAZ), transcriptional coactivators in the Hippo pathway, are crucial regulators of cancer development. Their nuclear localization and subsequent interaction with the transcriptional-enhanced associated domain (TEAD) transcription factors promote gene expression involved in cytoskeletal modulation and cell invasion. To separate the TEAD from its suppressor, the nuclear binding of unphosphorylated YAP/TAZ is required (Ortega et al., 2021). The activated complex can now control the expression of diverse factors responsible for the cytoskeleton modulation, fortifying the fibroblast matrix and enhancing the invasion of malignant cells (van Bree and Wilhelm, 2022). By inhibiting HMG-CoA reductase and disrupting the mevalonate pathway, statins significantly attenuate YAP/TAZ activity, offering a specific mechanism for targeting cancer cells (Zhu et al., 2021). Additionally, due to their unique properties, lipophilic statins might further affect YAP through their regulatory effects on RhoGTPases, which are key regulators of cell migration and invasion, thereby providing an additional mechanism for statins to target cancer cells (Hao et al., 2019).

### 3.2 Enhanced sensitivity to chemotherapy

Chemotherapy is a cornerstone of MB treatment for patients of all ages (Jackson and Packer, 2023). Adults also benefit from chemotherapy, with regimens like Packer and cisplatin-etoposide commonly utilized in Europe (Franceschi et al., 2021). However, the emergence of chemoresistance remains a significant hurdle. Statins have emerged as potential therapeutic agents to overcome this challenge (Tilija Pun and Jeong, 2021). The significance of these findings is underscored by studies that suggest synergistic effects between statins and certain chemotherapeutic drugs. Khandelwal et al. demonstrated that lovastatin enhanced the efficacy of methotrexate, tamoxifen, rapamycin, and doxorubicin in a yeast model system (Khandelwal Gilman et al., 2021). Talk *et al*. further supported these findings through a meta-analysis, highlighting the potential of statins to increase sensitivity to various chemotherapeutic agents, including cell cycle-specific and -nonspecific drugs, in preclinical models (Tulk et al., 2023).

Statins show promise in enhancing the chemosensitivity of cancer cells, including those in MB and glioblastoma multiforme (GBM). Studies have shown that statins can increase the sensitivity of MB cells to cisplatin, and in GBM cells treated with temozolomide (TMZ), they can enhance the drug's apoptotic effects (Fan et al., 2021, Rohrer et al., 2023). Importantly, statins can exert these effects through mechanisms independent of the mevalonate pathway, as observed in GBM cells exposed to TMZ (Sánchez et al., 2008). The current standard treatment for MB, involving surgery, craniospinal radiation, and high-dose chemotherapy, often leads to neurocognitive decline and increased mortality in recurrent cases (Ray et al., 2021, Rohrer et al., 2023, Newton, 2001). However, the potential of molecular targeted therapies, such as incorporating statins into conventional chemotherapy regimens, offer a promising strategy for MB. Statin co-therapy has the potential to not only improve treatment efficacy but also provide relief by mitigating the adverse effects of chemotherapy, particularly in high-risk patients (Rohrer et al., 2023).

As mentioned above, preclinical studies have demonstrated the promise of statins in MB therapy. Statins induce dose- and time-dependent cell death in various MB cell lines, leading to a significant increase in the population of apoptotic cells, and suggesting their potential as therapeutic agents. These findings suggest that combining statins with traditional anticancer drugs could offer a non-classical treatment approach for MB (Sheikholeslami et al., 2019). Furthermore, statins can act synergistically with conventional chemotherapy drugs. They sensitize MB cells during the late G1 phase, leading to enhanced inhibition of tumor cell proliferation when combined with chemotherapeutic agents like doxorubicin, paclitaxel, or topotecan (Shojaei et al., 2018, Rozados et al., 2005). Furthermore, population-based studies have also shown that statins provide a chemopreventive effect and improve survival rates in many types of malignancies. Nevertheless, the efficacy of this advantage has not been substantiated by clinical research (Altwairgi, 2015). In preclinical models, combining statins with chemotherapy agents such as doxorubicin, paclitaxel, and topotecan has demonstrated synergistic effects, resulting in enhanced inhibition of tumor cell proliferation compared to individual treatments. Moreover, this combination therapy has shown promising effects against hematopoietic tumors, including chronic lymphocytic leukemia, acute leukemia, and relapsed acute myelogenous leukemia (Henslee and Steele, 2018).

### 3.3 Reduced tumor recurrence

MB carries a high risk of recurrence (approximately 30 %) after standard therapy, leading to poor prognosis and limited treatment options (Ramaswamy et al., 2016). Emerging evidence suggests that statins, widely used for cholesterol reduction, may hold promise in reducing MB recurrence and improving patient outcomes. Statins exert their anti-tumor effects through multiple mechanisms, including disruption of cholesterol biosynthesis pathways essential for cancer cell survival (Gbelcova et al., 2017).

Preclinical studies support the potential of statins for MB therapy. Simvastatin induces apoptosis in various MB cell lines (Daoy, D283, D341) in a dose- and time-dependent manner, as evidenced by characteristic morphological changes. This is evidenced by the compression and division of cell nuclei, which are distinct features of apoptosis (Sheikholeslami et al., 2019). Notably, simvastatin exhibits cytotoxic activity against MB cells at concentrations ranging from 0.5 to 20 µM. *In vivo* studies have shown promise for combining statins with current MB therapies. For instance, simvastatin synergizes with hedgehog pathway inhibitors like vismodegib, significantly reducing tumor growth and MB cell proliferation in mouse models (Gordon et al., 2018). This suggests that statins may enhance the efficacy of existing treatments. Mechanistically, Sheikholeslami *et al*. demonstrated that simvastatin triggers apoptosis in MB cells bye activating caspases 3/7, 8, and 9, involving both intrinsic and extrinsic apoptotic pathways (Sheikholeslami et al., 2019). Furthermore, lovastatin-induced apoptosis appears to be independent of the p53 pathway, although p53 and p21WAF1 expression might contribute to the anti-proliferative effects of statins in certain MB cell lines (Wang and Macaulay, 2003). Hence, incorporating statins into medulloblastoma therapy regimens has great potential for decreasing tumor recurrence. Statins can trigger apoptosis in cancer cells, and when combined with hedgehog pathway inhibitors, they have the potential to create more powerful therapeutic approaches that not only stop tumor growth but also reduce the chances of cancer recurrence. Table 1 shows several functions in MB treatment.

**Table 1:** Several roles of statins in medulloblastoma therapy.

**Types of statins**	**Type of study**	**Dose**	**Effects**	**Ref.**
Simvastatin	*In vitro*	0–5–20 µM	• Prevented FPP and GGPP synthesis (by inhibiting HMGCR and subsequent suppression of MV pathway) • Induced caspase-dependent apoptosis. • Altered Bcl−2 expression (inducing apoptosis)	(Sheikholeslami et al., 2019)
	*In vitro* and *in vivo*	0–30 nM (*in vitro*); 200 mgmL (*in vivo*)	• Inhibited MB cell proliferation • Induced caspase-dependent apoptosis • Compromise Hh signaling • Enhanced the effect of vismodegib (50 mg/kg) in MB cell proliferation suppression	(Fan et al., 2021)
	*In vivo*	10 and 40 mg/kg daily for 2 weeks	• Decreased growth rate and size of tumor • Reduced cholesterol biosynthesis • Decreased Hh pathway activity	(Gordon et al., 2018)
	*In vitro*	0–5–20 µM	• Induce apoptosis in three various MB • Simvastatin-induced cell death is regulated through prenylation intermediates of the cholesterol metabolism pathway. • Alterations in the expression of regulator proteins involved in apoptosis, including Bax, Bcl−2, and Bcl-xl.	(Sheikholeslami et al., 2019)
Atrovastatin	*In vivo*	10 and 40 mg/kg	• Synergized with vismodegib in inhibiting MB cell proliferation • Decreased tumor size (at the dosage of 40 mg/kg)	(Fan et al., 2021)
Lovastatin	*In vitro*	20 µM	• Inhibited MB cell proliferation • Induced apoptosis	(Macaulay and Wang, 2014)
	*In vivo*	0.2–1 mg/kg	• Decreased cell proliferation • Reduced tumor growth • Increased survival in the mouse model	(Takwi et al., 2012)
	*In vitro*	20 µM	Lovastatin-induced apoptosis in MB is likely p53-independent, however p53 and p21WAF1 gene expression may also facilitate its anti-proliferative actions on select cell lines.	(Wang and Macaulay, 2003)

## 4 Clinical landscape and safety considerations in pediatrics

### 4.1 Clinical status of statin in MB

Currently, no clinical trials have specifically examined statins alone in medulloblastoma patients. However, there is an ongoing Phase I pediatric oncology trial that is assessing simvastatin combined with topotecan and cyclophosphamide for children with relapsed or refractory solid and CNS tumors, which might include cases of MB (ClinicalTrial.gov ID: NCT02390843). Evidence from preclinical studies using MB cell lines and mouse models reveals that simvastatin is capable of crossing the blood-brain barrier and diminishing tumor cell viability and invasion, with no apparent effects on bone growth—this is a crucial benefit when compared to Hedgehog inhibitors (Comer et al., 2023).

### 4.2 Safety profile of statins in children

Statins are commonly prescribed for pediatric patients diagnosed with familial hypercholesterolemia (FH), usually starting from the ages of 8–10 onward (Kaminska et al., 2016, Kavey et al., 2020, Motkowski et al., 2023). Meta-analyses and randomized controlled trials indicate a decrease in LDL-C by 20–50 %, without significant negative impacts on growth, development, liver function, or musculoskeletal health during study durations of up to two years (Kavey et al., 2020, Vuorio et al., 2019). A retrospective multicenter study conducted in real-world settings, which included almost 300 children (average age approximately 12 years, median follow-up of about 2.7 years), demonstrated consistent lipid reduction and outstanding tolerability, with no instances of treatment discontinuation attributed to safety issues (Kavey et al., 2020). Recently, a 20-year observational cohort study provided evidence for the ongoing effectiveness of statin therapy in children with familial hypercholesterolemia (FH), showing no detrimental effects on their physical development (Motkowski et al., 2023).

### 4.3 Key safety consideration in MB

Despite the positive data emerging from pediatric FH populations, there is a deficiency of direct evidence in the field of pediatric neuro-oncology. Preclinical models illustrate that simvastatin effectively inhibits MB growth in juvenile mice without negatively impacting bone development, likely due to its minimal bone penetration and the suppression of Hedgehog signaling in the tumor cells (Fan et al., 2021, Comer et al., 2023). Nonetheless, there are still potential risks present in the clinical setting: 1) Bone growth and development—Hedgehog signaling plays a crucial role in skeletal maturation; however, the accumulation of statins in bone seems to be minimal according to murine studies (Zaky et al., 2023, Fan et al., 2021). 2) Hepatic or muscular toxicity, particularly when paired with cytotoxic chemotherapy (Jose, 2016).

### 4.4 Recommendations for future clinical trials

To ensure a responsible evaluation of statins in pediatric MB, future Phase I/II trials are required to feature. For instance, Regular monitoring of liver transaminases (ALT/AST) and CK should be conducted at baseline, 1–2 months after treatment initiation, and then every 3–6 months during the first year, with a shift to every 6–12 months thereafter—CK testing should be performed immediately if muscle symptoms present themselves (Spoiala et al., 2024, Fiorentino and Chiarelli, 2023), and assessment of growth metrics, such as height, bone age imaging, and growth indicators. Dosing decisions are based on pediatric pharmacokinetics, extrapolated from safe exposure levels in preclinical studies.

## 5 Future directions and implications

Recent attention has been directed towards the potential anticancer properties of cholesterol-lowering agents known as 'statins' (Sheikholeslami et al., 2019). While preclinical studies and early-phase clinical trials suggest promising potential for statins in MB treatment, further research is necessary to fully translate these findings into clinical practice. This would involve analyzing the genetic and molecular characteristics of tumors to determine the appropriate usage of statins in certain groups of patients (Sheikholeslami et al., 2019).

### 5.1 Optimizing treatment strategies

Large-scale, well-designed clinical trials are crucial to confirm the efficacy and safety of statins in MB patients. These trials should aim to identify optimal dosing schedules that maximize therapeutic benefit while minimizing side effects. Additionally, a personalized medicine approach is necessary due to the diverse responses observed across MB subtypes. Genomic and molecular profiling of tumors should be utilized to guide statin treatment decisions and identify patient populations most likely to benefit.

### 5.2 Combination therapies and synergistic effects

Investigating the synergistic effects of statins with current MB therapies, such as Hedgehog pathway inhibitors or immune checkpoint inhibitors, holds promise for developing more potent and durable treatment regimens. Further exploration of the ability of statin to modulate the TME and enhance immunotherapeutic responses could open new avenues for MB therapy.

### 5.3 Delivery strategies and bioavailability enhancement

Developing nanoparticle-based drug delivery systems or formulations that enhance blood-brain barrier permeability could significantly improve statin bioavailability and therapeutic efficacy in MB.

### 5.4 Unveiling mechanisms and expanding applications

As our understanding of statin's pleiotropic effects in cancer biology expands, their potential application may extend beyond MB to other malignancies. Continuing research is essential to fully elucidate the mechanisms by which statins enhance chemotherapy sensitivity in MB.

### 5.5 Repurposing for global health impact

Given their established safety profile and widespread availability, statins represent a cost-effective and readily accessible treatment option (Suh et al., 2018), particularly in resource-limited settings.

In order to optimize the use of statins in MB treatment regimens and to completely understand how they boost chemotherapy sensitivity, further research and clinical studies are needed (Vuu et al., 2023). By addressing these future directions, researchers can unlock the full potential of statins for improving MB treatment outcomes. Additionally, their repurposing as anticancer agents in other tumor types offers exciting possibilities for broader clinical impact.

## 6 Conclusion

Statins, widely prescribed cholesterol-lowering drugs, have garnered significant interest for their potential anticancer properties, particularly in the context of MB treatment. Emerging evidence from preclinical studies and clinical investigations suggests that statins may offer a promising therapeutic approach for managing this aggressive pediatric brain tumor. The primary mechanism by which statins exert their anti-tumor effects in MB involves the inhibition of the mevalonate pathway. By targeting the rate-limiting enzyme HMG-CoA reductase, statins deplete cellular pools of isoprenoid intermediates, including farnesyl pyrophosphate and geranylgeranyl pyrophosphate, which are essential for the proper localization and function of signaling proteins involved in cell proliferation and survival. Notably, this pathway plays a crucial role in the sonic hedgehog (SHH) subgroup of MB, where aberrant activation of the hedgehog signaling cascade drives tumorigenesis. By reducing cholesterol levels, statins can effectively suppress the hedgehog pathway, thereby inhibiting tumor growth and proliferation in SHH-driven MB. Beyond their effects on the MV pathway, statins have been shown to induce apoptosis in MB cells through both intrinsic and extrinsic pathways. This pro-apoptotic activity involves the modulation of key regulators, such as caspases and members of the Bcl-2 family. Interestingly, statins exhibit a selective ability to trigger apoptosis in cancer cells while sparing normal cells, potentially offering a favorable therapeutic index, a crucial consideration in pediatric MB treatment. Furthermore, statins have demonstrated the capacity to enhance the sensitivity of MB cells to conventional chemotherapeutic agents, such as cisplatin and temozolomide. This chemo-sensitizing effect is mediated through various mechanisms, including cell cycle modulation, induction of oxidative stress, and disruption of survival signaling pathways. By potentiating the cytotoxic effects of chemotherapy, statins offer the potential for more effective and durable treatment responses. Moreover, statins modulate the activity of key transcriptional regulators, such as YAP/TAZ, which govern cellular processes essential for cancer development and metastasis. Additionally, investigating the synergistic effects of statins with other targeted therapies, such as hedgehog pathway inhibitors or immunotherapies, could lead to the development of more potent and durable treatment strategies. In summary, statins represent a promising repurposed therapeutic approach for MB, offering a multifaceted mechanism of action that targets key pathways involved in tumor growth, survival, and resistance mechanisms. By leveraging their pleiotropic effects and exploring combination strategies, statins hold the potential to improve treatment outcomes and quality of life for patients with this devastating childhood cancer.

## Funding

The author(s) declare that no financial support was received for the research, authorship, and/or publication of this article.

## CRediT authorship contribution statement

**Amir Modarresi Chahardehi:** Writing – original draft. **Aida Naseri:** Writing – original draft, Supervision. **Esfandiar Mali:** Writing – original draft. **Leili Ziaei:** Writing – original draft. **Fatemeh Moradi:** Writing – original draft. **Fatemeh Teimourpour:** Writing – original draft. **Leila Tajik:** Writing – original draft. **Hossein Motedayyen:** Writing – review & editing. **Mohammad Saeed Soleimani Meigoli:** Visualization. **Reza Nasiri:** Validation. **Reza Arefnezhad:** Writing – review & editing, Supervision. **Fatemeh Rezaei-Tazangi:** Writing – original draft.

## Declaration of Competing Interest

The authors declare that there is no conflict of interests

## References

[bib1] Ahmadi M. (2020). Pleiotropic effects of statins: a focus on cancer. Biochim Biophys. Acta Mol. Basis Dis..

[bib2] Alizadehasl A. (2024). Lipid-lowering drugs and cancer: an updated perspective. Pharm. Rep..

[bib3] Aljoghaiman M., Taha M.S., Abdulkader M.M. (2018). Cerebellar medulloblastoma in Middle-to-Late adulthood. Case Rep. Pathol..

[bib4] Alrosan A.Z. (2023). The effects of statin therapy on brain tumors, particularly glioma: a review. Anticancer Drugs.

[bib5] Altwairgi A.K. (2015). Statins are potential anticancerous agents (review). Oncol. Rep..

[bib6] Ando S., Araki K. (2022). CD8 T-cell heterogeneity during T-cell exhaustion and PD-1-targeted immunotherapy. Int Immunol..

[bib7] Bebber C.M. (2020). Ferroptosis in cancer cell biology. Cancers (Basel).

[bib8] Di Bello E. (2020). The innovative potential of statins in cancer: new targets for new therapies. Front. Chem..

[bib9] van Bree N.F., Wilhelm M. (2022). The tumor microenvironment of medulloblastoma: an intricate multicellular network with therapeutic potential. Cancers.

[bib10] Breitling J., Aebi M. (2013). N-linked protein glycosylation in the endoplasmic reticulum. Cold Spring Harb. Perspect. Biol..

[bib11] Choe E.J. (2022). Atorvastatin enhances the efficacy of immune checkpoint therapy and suppresses the cellular and extracellular vesicle PD-L1. Pharmaceutics.

[bib12] Chou C.-W. (2019). Therapeutic effects of statins against lung adenocarcinoma via p53 mutant-mediated apoptosis. Sci. Rep..

[bib13] Comer C., Michod D., Niklison-Chirou M.V. (2023). Simvastatin inhibits medulloblastoma cell migration via targeting the mevalonate pathway. NeuroOncol..

[bib14] Corcoran R.B., Scott M.P. (2006). Oxysterols stimulate sonic hedgehog signal transduction and proliferation of medulloblastoma cells. Proc. Natl. Acad. Sci. USA.

[bib15] Cotter J.A., Hawkins C. (2022). Medulloblastoma: WHO 2021 and beyond. Pedia Dev. Pathol..

[bib16] Fang F.Y. (2022). New developments in the pathogenesis, therapeutic targeting, and treatment of pediatric medulloblastoma. Cancers (Basel).

[bib17] Fan Q. (2021). Statins repress hedgehog signaling in medulloblastoma with no bone toxicities. Oncogene.

[bib18] Ferguson S., Lesniak M.S. (2005). Percival bailey and the classification of brain tumors. Neurosurg. Focus.

[bib19] Filaferro L. (2024). Are statins onco- suppressive agents for every type of tumor? A systematic review of literature. Expert Rev. Anticancer Ther..

[bib20] Fiorentino R., Chiarelli F. (2023). Statins in children, an update. Int J. Mol. Sci..

[bib21] Franceschi E. (2021). How we treat medulloblastoma in adults. ESMO Open.

[bib22] Gajjar A. (2021). Outcomes by clinical and molecular features in children with medulloblastoma treated with Risk-Adapted therapy: results of an international phase III trial (SJMB03). J. Clin. Oncol..

[bib23] Gbelcova H. (2017). Isoprenoids responsible for protein prenylation modulate the biological effects of statins on pancreatic cancer cells. Lipids Health Dis..

[bib24] Gobel A. (2024). Overcoming statin resistance in prostate cancer cells by targeting the 3-hydroxy-3-methylglutaryl-CoA-reductase. Biochem Biophys. Res Commun..

[bib25] Göbel A. (2020). Cholesterol and beyond - the role of the mevalonate pathway in cancer biology. Biochim Biophys. Acta Rev. Cancer.

[bib26] Gordon R.E. (2018). Statins synergize with hedgehog pathway inhibitors for treatment of medulloblastoma. Clin. Cancer Res.

[bib27] Gregory T.A. (2023). Characterization of recurrence patterns and outcomes of medulloblastoma in adults: the university of texas MD anderson cancer center experience. Neurooncol Adv..

[bib28] Guerra B. (2021). The mevalonate pathway, a metabolic target in cancer therapy. Front Oncol..

[bib29] Gwynne W.D. (2022). Cancer-selective metabolic vulnerabilities in MYC-amplified medulloblastoma. Cancer Cell.

[bib30] Hao F. (2019). Lipophilic statins inhibit YAP nuclear localization, co-activator activity and colony formation in pancreatic cancer cells and prevent the initial stages of pancreatic ductal adenocarcinoma in KrasG12D mice. PLoS One.

[bib31] Henslee A.B., Steele T.A. (2018). Combination statin and chemotherapy inhibits proliferation and cytotoxicity of an aggressive natural killer cell leukemia. Biomark. Res.

[bib32] Huang S.W. (2020). Lovastatin-mediated MCF-7 cancer cell death involves LKB1-AMPK-p38MAPK-p53-survivin signalling cascade. J. Cell. Mol. Med..

[bib33] Jackson K., Packer R.J. (2023). Recent advances in pediatric medulloblastoma. Curr. Neurol. Neurosci. Rep..

[bib34] Jaganjac M. (2021). Lipid peroxidation in brain tumors. Neurochem. Int..

[bib35] Jiang J. (2022). Hedgehog signaling mechanism and role in cancer. Semin Cancer Biol..

[bib36] Jiang W. (2021). Statins: a repurposed drug to fight cancer. J. Exp. Clin. Cancer Res.

[bib37] Jose J. (2016). Statins and its hepatic effects: newer data, implications, and changing recommendations. J. Pharm. Bioallied Sci..

[bib38] Juraschka K., Taylor M.D. (2019). Medulloblastoma in the age of molecular subgroups: a review. J. Neurosurg. Pedia.

[bib39] Kabir T.F. (2020). Immunotherapy for medulloblastoma: current perspectives. Immunotargets Ther..

[bib40] Kaminska E. (2016). Treatment with statins in children with familial hypercholesterolemia. Dev. Period Med.

[bib41] Kansal V. (2023). Statin drugs enhance responses to immune checkpoint blockade in head and neck cancer models. J. Immunother. Cancer.

[bib42] Kavey R.W. (2020). Effectiveness and safety of statin therapy in children: a Real-World clinical practice experience. CJC Open.

[bib43] Khalaf K. (2021). Aspects of the tumor microenvironment involved in immune resistance and drug resistance. Front. Immunol..

[bib44] Khalifeh M. (2021). Statins as anti-pyroptotic agents. Arch. Med Sci..

[bib45] Khandelwal Gilman K.A. (2021). Complex interactions of lovastatin with 10 chemotherapeutic drugs: a rigorous evaluation of synergism and antagonism. BMC Cancer.

[bib46] Kimura S. (2023). Cholesterol in the ciliary membrane as a therapeutic target against cancer. Front Mol. Biosci..

[bib47] Konstantinopoulos P.A., Karamouzis M.V., Papavassiliou A.G. (2007). Post-translational modifications and regulation of the RAS superfamily of GTPases as anticancer targets. Nat. Rev. Drug Discov..

[bib48] Kool M. (2012). Molecular subgroups of medulloblastoma: an international meta-analysis of transcriptome, genetic aberrations, and clinical data of WNT, SHH, group 3, and group 4 medulloblastomas. Acta Neuropathol..

[bib49] Lasuncion M.A. (2022). Cell cycle dependence on the mevalonate pathway: role of cholesterol and non-sterol isoprenoids. Biochem Pharm..

[bib50] Liu C. (2023). New insights into the therapeutic potentials of statins in cancer. Front Pharm..

[bib51] Li B. (2022). Lipid raft involvement in signal transduction in cancer cell survival, cell death and metastasis. Cell Prolif..

[bib52] Loffler L., Gogenur I., Gogenur M. (2024). Correlations between preoperative statin treatment with short- and long-term survival following colorectal cancer surgery: a propensity score-matched national cohort study. Int J. Colorectal Dis..

[bib53] Louis D.N. (2021). The 2021 WHO classification of tumors of the central nervous system: a summary. Neuro Oncol..

[bib54] Louis D.N. (2016). The 2016 world health organization classification of tumors of the central nervous system: a summary. Acta Neuropathol..

[bib55] Macaulay R.J.B., Wang W. (2014). Cell-Cycle gene expression in Lovastatin-Induced medulloblastoma apoptosis. Can. J. Neurol. Sci. / J. Can. Des. Sci. Neurol..

[bib56] Martin-Rubio P. (2022). Metabolic determinants of stemness in medulloblastoma. World J. Stem Cells.

[bib57] McGregor G.H. (2020). Targeting the metabolic response to Statin-Mediated oxidative stress produces a synergistic antitumor response. Cancer Res.

[bib58] McKenzie B.A., Dixit V.M., Power C. (2020). Fiery cell death: pyroptosis in the central nervous system. Trends Neurosci..

[bib59] Mduma E., Awuor A., Lugina E.L. (2022). Adult medulloblastoma: a case report. J. Med Case Rep..

[bib60] Menyhart O., Gyorffy B. (2020). Molecular stratifications, biomarker candidates and new therapeutic options in current medulloblastoma treatment approaches. Cancer Metastas.. Rev..

[bib61] Mir S.E. (2017). Trimethylation of H3K27 during human cerebellar development in relation to medulloblastoma. Oncotarget.

[bib62] Morofuji Y. (2022). Beyond Lipid-Lowering: effects of statins on cardiovascular and cerebrovascular diseases and cancer. Pharm. (Basel).

[bib63] Motkowski R. (2023). Efficacy and safety of statin treatment in children with familial hypercholesterolemia: outcomes of 20 years of experience. J. Clin. Med..

[bib64] Newton H.B. (2001). Review of the molecular genetics and chemotherapeutic treatment of adult and paediatric medulloblastoma. Expert Opin. Invest. Drugs.

[bib65] Okonechnikov K. (2023). Comparison of transcriptome profiles between medulloblastoma primary and recurrent tumors uncovers novel variance effects in relapses. Acta Neuropathol. Commun..

[bib66] Okubo K. (2020). Fluvastatin potentiates anticancer activity of vorinostat in renal cancer cells. Cancer Sci..

[bib67] de Oliveira F., Landeiro J.A., de Castro I. (2018). Adult hemispheric cerebellar medulloblastoma. Surg. Neurol. Int.

[bib68] Orr B.A. (2020). Pathology, diagnostics, and classification of medulloblastoma. Brain Pathol..

[bib69] Ortega Á. (2021). The YAP/TAZ signaling pathway in the tumor microenvironment and carcinogenesis: current knowledge and therapeutic promises. Int. J. Mol. Sci..

[bib70] Palsuledesai C.C., Distefano M.D. (2015). Protein prenylation: enzymes, therapeutics, and biotechnology applications. ACS Chem. Biol..

[bib71] Patay Z. (2015). MR imaging characteristics of Wingless-Type-Subgroup pediatric medulloblastoma. AJNR Am. J. Neuroradiol..

[bib72] Patel K.K., Sehgal V.S., Kashfi K. (2022). Molecular targets of statins and their potential side effects: not all the glitter is gold. Eur. J. Pharm..

[bib73] Patmore D.M. (2020). DDX3X suppresses the susceptibility of hindbrain lineages to medulloblastoma. Dev. Cell.

[bib74] Phoenix T.N. (2016). Medulloblastoma genotype dictates blood brain barrier phenotype. Cancer Cell.

[bib75] Pokhrel R.H. (2021). AMPK promotes antitumor immunity by downregulating PD-1 in regulatory t cells via the HMGCR/p38 signaling pathway. Mol. Cancer.

[bib76] Raffel C. (2004). Medulloblastoma: molecular genetics and animal models. Neoplasia.

[bib77] Ramaswamy V. (2013). Recurrence patterns across medulloblastoma subgroups: an integrated clinical and molecular analysis. Lancet Oncol..

[bib78] Ramaswamy V. (2016). Medulloblastoma subgroup-specific outcomes in irradiated children: who are the true high-risk patients?. Neuro Oncol..

[bib79] Rawal Z.D. (2020). Medulloblastoma under siege: genetic and molecular dissection concerning recent advances in therapeutic strategies. J. Pedia Neurosci..

[bib80] Ray S. (2021). Subgroup-Specific diagnostic, prognostic, and predictive markers influencing pediatric medulloblastoma treatment. Diagn. (Basel).

[bib81] Roberts J.Z., Crawford N., Longley D.B. (2022). The role of ubiquitination in apoptosis and necroptosis. Cell Death Differ..

[bib82] Rohrer K.A. (2023). STAT3 inhibition attenuates MYC expression by modulating Co-Activator recruitment and suppresses medulloblastoma tumor growth by augmenting cisplatin efficacy in vivo. Cancers (Basel).

[bib83] Rozados V.R. (2005). Lovastatin enhances in vitro radiation-induced apoptosis of rat B-cell lymphoma cells. J. Exp. Clin. Cancer Res.

[bib84] Sahebkar A. (2023). Ferroptosis, a new pathogenetic mechanism in cardiometabolic diseases and cancer: is there a role for statin therapy?. Metabolism.

[bib85] Sánchez C.A. (2008). Statin-induced inhibition of MCF-7 breast cancer cell proliferation is related to cell cycle arrest and apoptotic and necrotic cell death mediated by an enhanced oxidative stress. Cancer Invest.

[bib86] Santoni M. (2022). Statins and renal cell carcinoma: antitumor activity and influence on cancer risk and survival. Crit. Rev. Oncol. Hematol..

[bib87] Sheikholeslami K. (2019). Simvastatin induces apoptosis in medulloblastoma brain tumor cells via mevalonate cascade prenylation substrates. Cancers (Basel).

[bib88] Shojaei S. (2018). Statins: a new approach to combat temozolomide chemoresistance in glioblastoma. J. Invest. Med.

[bib89] Singh P., Lim B. (2022). Targeting apoptosis in cancer. Curr. Oncol. Rep..

[bib90] Smoll N.R., Drummond K.J. (2012). The incidence of medulloblastomas and primitive neurectodermal tumours in adults and children. J. Clin. Neurosci..

[bib91] Spoiala E.L. (2024). Statins—Beyond their use in hypercholesterolemia: focus on the pediatric population. Children.

[bib92] Sturm D. (2016). New brain tumor entities emerge from molecular classification of CNS-PNETs. Cell.

[bib93] Suh D.C. (2018). Comparative effectiveness of lipid-lowering treatments to reduce cardiovascular disease. Expert Rev. Pharm. Outcomes Res.

[bib94] Takwi A.A. (2012). A statin-regulated microRNA represses human c-Myc expression and function. EMBO Mol. Med..

[bib95] Tang J.Y., So P.L. (2007). And E.H. Epstein, jr., *novel hedgehog pathway targets against basal cell carcinoma.*. Toxicol. Appl. Pharm..

[bib96] Le Teuff G. (2020). Phase II study of temozolomide and topotecan (TOTEM) in children with relapsed or refractory extracranial and central nervous system tumors including medulloblastoma with post hoc Bayesian analysis: a european ITCC study. Pedia Blood Cancer.

[bib97] Tilija Pun N., Jeong C.-H. (2021). Statin as a potential chemotherapeutic agent: current updates as a monotherapy, combination therapy, and treatment for anti-cancer drug resistance. Pharmaceuticals.

[bib98] Tripathi S., Gupta E., Galande S. (2024). Statins as anti-tumor agents: a paradigm for repurposed drugs. Cancer Rep (Hoboken).

[bib99] Tulk A., Watson R., Erdrich J. (2023). The influence of statin use on chemotherapeutic efficacy in studies of mouse models: a systematic review. Anticancer Res.

[bib100] Vuorio A. (2019). Statins for children with familial hypercholesterolemia. Cochrane Database Syst. Rev..

[bib101] Vuu Y.M., Kadar Shahib A., Rastegar M. (2023). The potential therapeutic application of simvastatin for brain complications and mechanisms of action. Pharmaceuticals.

[bib102] Waller D.D., Park J., Tsantrizos Y.S. (2019). Inhibition of farnesyl pyrophosphate (FPP) and/or geranylgeranyl pyrophosphate (GGPP) biosynthesis and its implication in the treatment of cancers. Crit. Rev. Biochem Mol. Biol..

[bib103] Wang W., Macaulay R.J. (2003). Cell-cycle gene expression in lovastatin-induced medulloblastoma apoptosis. Can. J. Neurol. Sci..

[bib104] Wen J., Hadden M.K. (2021). Medulloblastoma drugs in development: current leads, trials and drawbacks. Eur. J. Med Chem..

[bib105] Wong J.V.S., Fatehi Hassanabad A. (2021). Targeting the mevalonate pathway for treating esophageal cancer. J. Gastrointest. Cancer.

[bib106] Xiao X. (2017). Cholesterol modification of smoothened is required for hedgehog signaling. Mol. Cell.

[bib107] Yao X. (2021). Simvastatin induced ferroptosis for triple-negative breast cancer therapy. J. nanobiotechnology.

[bib108] Yin Y. (2018). Simvastatin inhibits sonic hedgehog signaling and stemness features of pancreatic cancer. Cancer Lett..

[bib109] You H.M. (2023). Pyroptosis: shedding light on the mechanisms and links with cancers. Front Immunol..

[bib110] Zaky M.Y. (2023). Unraveling the anticancer potential of statins: mechanisms and clinical significance. Cancers (Basel).

[bib111] Zhang J., Wang T. (2021). Immune cell landscape and immunotherapy of medulloblastoma. Pedia Invest..

[bib112] Zhou Z. (2023). Research progress in molecular pathology markers in medulloblastoma. Explor Target Antitumor Ther..

[bib113] Zhu P.F. (2021). Targeting the tumor microenvironment: a literature review of the novel Anti-Tumor mechanism of statins. Front Oncol..

